# Treatment of tympanic membrane perforation using bacterial cellulose: a randomized controlled trial^☆^^☆☆^

**DOI:** 10.1016/j.bjorl.2015.03.015

**Published:** 2015-09-08

**Authors:** Fábio Coelho Alves Silveira, Flávia Cristina Morone Pinto, Sílvio da Silva Caldas Neto, Mariana de Carvalho Leal, Jéssica Cesário, José Lamartine de Andrade Aguiar

**Affiliations:** a Graduate Program in Surgery, Department of Surgery, Universidade Federal de Pernambuco (UFPE), Recife, PE, Brazil; b Nucleus of Experimental Surgery, Universidade Federal de Pernambuco (UFPE), Recife, PE, Brazil; c Service of Otolaryngology, Universidade Federal de Pernambuco (UFPE), Recife, PE, Brazil; d Universidade Federal de Pernambuco (UFPE), Recife, PE, Brazil; e Medical Course, Universidade Federal de Pernambuco (UFPE), Recife, PE, Brazil

**Keywords:** Tympanic membrane perforation, Biopolymer, Bacterial cellulose, Perfuração da membrana timpânica, Biopolímero, Celulose bacteriana

## Abstract

**Introduction:**

Promising treatments for tympanic membrane perforation closure have been studied. Therapies derived from tissue engineering probably eliminate the need for conventional surgery. Bacterial cellulose is presented as an alternative that is safe, biocompatible, and has low toxicity.

**Objectives:**

To investigate the effect on healing of direct application of a bacterial cellulose graft on the tympanic membrane compared to the conventional approach with autologous fascia.

**Methods:**

Randomized controlled trial. Forty patients with tympanic membrane perforations secondary to chronic otitis media were included, and were randomly assigned to an experimental group (20), treated with a bacterial cellulose graft (BC) and control group (20), treated with autologous temporal fascia (fascia). We evaluated the surgical time, hospital stay, time of epithelialization and the rate of tympanic perforation closure. Hospital costs were compared. The statistical significance level accepted was established at *p* < 0.05.

**Results:**

The closure of perforations was similar in both groups. The average operation time in the fascia group was 76.50 min *versus* 14.06 min bacterial cellulose in the group (*p* = 0.0001). The hospital cost by the Brazilian public health system was R$ 600.00 for the bacterial cellulose group, and R$ 7778.00 for the fascia group (*p* = 0.0001).

**Conclusion:**

Bacterial cellulose grafts promoted the closure of the tympanic membrane perforations, and were demonstrated to be innovative, effective, safe, minimally invasive, efficacious and to have a very low cost.

## Resumo

**Introdução:**

Tratamentos promissores para o fechamento da perfuração da membrana timpânica vêm sendo estudados. Terapias provenientes de engenharia de tecidos provavelmente eliminarão a necessidade de uma intervenção cirúrgica convencional. A celulose bacteriana apresenta-se como uma alternativa por ser segura, de baixa toxicidade, biocompatível.

**Objetivos:**

Investigar o efeito da aplicação direta do enxerto da celulose bacteriana na cicatrização de perfurações da membrana timpânica, comparado ao procedimento convencional com fáscia autóloga.

**Método:**

Incluíram-se 40 pacientes com perfuração da membrana timpânica por otite média crônica simples. Randomizados de 1 a 40, onde os ímpares (20) foram tratados com enxerto de celulose bacteriana (CB), e os pares (20), com enxerto de fáscia temporal autóloga (fáscia). Estudo clínico controlado e randomizado. O tempo cirúrgico e de hospitalização foram o tempo de epitelização e custos hospitalares.

**Resultados:**

O fechamento das perfurações foi semelhante nos dois grupos. O tempo médio da cirurgia no grupo fáscia foi de 76,50 minutos e de 14,06 minutos no grupo com celulose bacteriana (p = 0,0001). O custo hospitalar pela tabela do SUS foi de R$ 600,00 para o grupo CB e R$ 7.778,00 para o grupo fáscia (p = 0,0001).

**Conclusão:**

A celulose bacteriana promoveu o fechamento da perfuração do tímpano, mostrando-se inovador, seguro, eficaz, efetivo, minimamente invasivo e de baixo custo.

## Introduction

Promising treatments for closure of tympanic membrane perforation (TMP) have been studied, in search of outpatient, minimally invasive procedures that are effective, safe, affordable and technically feasible.^1^, ^2^, ^3^, ^4^, ^5^ Among some innovative alternatives, the use of gelfoam™ and atelocollagen™ stand out, in association with fibroblast growth factor (β-FGF),^1^, ^2^, ^3^, ^4^ autologous serum, and chitin membranes.^5^

The establishment of a therapy developed from tissue engineering for treatment of TMP will probably eliminate the need for conventional surgery. However, it is critical to understand the factors that contribute to the success or failure of TMP treatment.^4^

An alternative material is cellulosic polysaccharide, obtained by bacterial synthesis. In previous studies, cellulosic polysaccharide proved to be a safe, low-toxicity,^6^ biocompatible^7^ product, with the ability to encourage cellular growth and differentiation – a feature that is promising for tissue engineering.^8^ Preclinical and clinical studies have demonstrated that this biomaterial was effective functioning as a mechanical barrier and as an adjunct in the treatment of ulcerative lesions^9^ and surgical wounds.^10^

The objective of this study was to investigate the effect of direct application of a bacterial cellulose (BC) graft on the healing of tympanic membrane perforations, compared with a conventional procedure with autologous fascia.

## Method

Forty patients with tympanic membrane perforations caused by otitis media were enrolled in a randomized controlled clinical study of spontaneous demand at Otolaryngology Service in a teaching hospital in Pernambuco state, Brazil, from 2013 to 2014. Patients with marginal, damp or cholesteatomatous perforations were excluded. Patients were randomly allocated to two groups: 20 in an experimental group, who were treated with bacterial cellulose membrane graft, and 20 controls treated conventionally with autologous fascia graft.

This study was approved by the Ethics Committee on Research, Health Sciences Center, Universidade Federal de Pernambuco, under CAAE 21109913.7.0000.5208, Opinion CEP/CONEP No. 527.461 of December 18, 2013.

### Bacterial cellulose graft

Bacterial cellulose grafts were manufactured and supplied by Polisa™, a Sugar Cane Experimental Station, Carpina City, Universidade Federal Rural de Pernambuco, Brazil.^11^

### Technical procedures

Patients included in control group underwent miringoplasty with temporal fascia graft, performed under general anesthesia, according to standard operating procedures for this surgery. The graft of fascia was applied medial to the tympanic remants under the handle of malleus and middle ear, and held in position with Gelfoam™ fragments. At the end of the procedure, the incision was sutured in anatomical planes, and a pressure dressing was applied. The patient remained in the hospital until the next day. At the time of hospital discharge, cephalexin 500 mg, orally, four times daily for 7 days was prescribed, and the patient was instructed to return to his/her activities after 8–15 days.

For patients in the experimental group, the procedure was performed under local anesthesia with infiltration of xylocaine (2% solution) 5.0 ml with vasoconstrictor, divided into two parts: 2.5 ml for external application and 2.5 ml into the external auditory canal. The perforation edges were scarified, and then a bacterial cellulose membrane was placed over the perforation, laterally to the tympanic remains. The membrane was held in place by self-adhesion. The patient was discharged immediately after the procedure, being instructed to return to his/her activities without restrictions. Antibiotics were not prescribed.

### Outcomes evaluated

#### Clinical outcomes

In both groups, the following variables were evaluated: surgical time, hospital stay, time for epithelialization, TMP closing rate in *t*^0^ = 15 days, *t*^1^ = 30 days and *t*^2^ = 60 days; the impedance audiometry curve 60 days post-treatment, and adverse events.

Hospital costs were analyzed separately for use of BC (experimental group) *versus* temporal fascia (control group). These costs were estimated according to the table of the Brazilian Unified Health System (SUS) from Ministry of Health, 2007, taking into account: for autologous fascia, tympanoplasty (uni/bilateral) (code: 04.04.01.035-0), surgical specialty of medium complexity; the assigned value includes one (1) day of hospitalization (R$ 388.94 per patient); for BC grafting, a grade II dressing (code: 04.01.01.001-5), surgical specialty of medium complexity, with no inclusion of hospital stay (R$ 30.00 per patient).

Tympanometry: The evaluation of tympanic membrane mobility was obtained based on the impedance chart, considering air pressure (marked on the *X* axis in decaPascal, daPaX) and admittance (on the *Y* axis, daPaY in ml).^12^

*Effectiveness*: Effectiveness represents the relative reduction of risk or of negative outcome (TMP closure) obtained with the intervention (in this case, the use of BC). Relative risk [RR = R(BC)/R (fascia)] was calculated, followed by absolute reduction in risk (ARR = [R(fascia) − R(BC)] × 100) and effectiveness [EF = (1 − RR) × 100] calculations. When the risk is equal in both groups, RR = 1. If the risk for intervention group is lower than the risk for control group, RR < 1; otherwise, RR > 1.

Parametric continuous variables were compared using Student's *t* test, while scores were compared using the chi-squared test. Mann–Whitney test was used to evaluate the sum of hospital costs. A 95% confidence interval was used, and the statistical significance was set at *p* ≤ 0.05. Statistical analysis was performed using GraphPad Prism 5.0 software (GraphPad Software Inc., USA).

## Results

A total of 40 patients underwent treatment for tympanic membrane perforation; 20 received a BC graft (30% men and 70% women); and 20 – the control group – received an autologous fascia graft (40% men and 60% women). The mean age of the groups was 38.15 ± 12.63, and 34.5 ± 10.16 years, respectively (Table 1).

**Table 1:** Outcomes evaluated between groups treated with bacterial cellulose and autologous fascia grafts, for treatment of perforated tympanic membrane.

Outcomes	Type of graft		*p*-Value
	BC	Fascia	
*N*	20	20	–

*Gender*			
Male	06 (30%)	08 (40%)	0.5073^a^
Female	14 (70%)	12 (60%)	

*Age*	38.15 ± 12.63	34.5 ± 10.16	0.3204^b^

*TMP location*			
Right ear	7 (35.0%)	11(55.0%)	0.2036^a^
Left ear	13 (65.0%)	9 (45.0%)	

*TMP size*			
Small	14 (70.0%)	14 (70.0%)	1.000^a^
Medium	6 (30.0%)	6 (30.0%)	

*Surgical time* (min)	14.06 ± 5.23	76.50 ± 17.92	<0.0001^b^^*^

*TMP closure*			
General	18 (90.0%)	16 (80.0%)	0.3758^a^

*By size*			
Small	14 (100%)	13 (92.9%)	0.6264^a^
Medium	04 (66.7%)	03 (50%)	0.5582^a^

*Hospital cost estimate*^c^			
Per patient	30.00	388.94	
Total	600.00	7778.80	<0.0001^c^

*Risk and efficacy analysis*			
RR%	0.5		0.3758^a^
ARR	10%		–
Efficacy	50%		

BC, Bacterial cellulose; TMP, Tympanic membrane perforation; RR, Relative risk; ARR, Absolute risk reduction.

Values in mean ± SD and *n* (%).

a Qui-squared test.

b Student's *t* test, significant if (*) *p* ≤ 0.05.

c Hospital cost estimate, according to the table of costs for surgical procedures of the Brazilian Unified Health System (SUS); values in Reais (R$). Mann–Whitney test.

In the group of patients who received BC graft, 65% of TMPs were in the left ear, while in the group that received an autologous fascia graft, most (55%) TMPs were in the right ear. Perforations were more often of small size, accounting for 70% of cases in each group. Closure occurred in all small perforations treated by BC graft, when compared with fascia graft (92.9%). More than half (66.6%) of medium-sized perforations closed with BC graft (Table 1).

**Figure 1:**
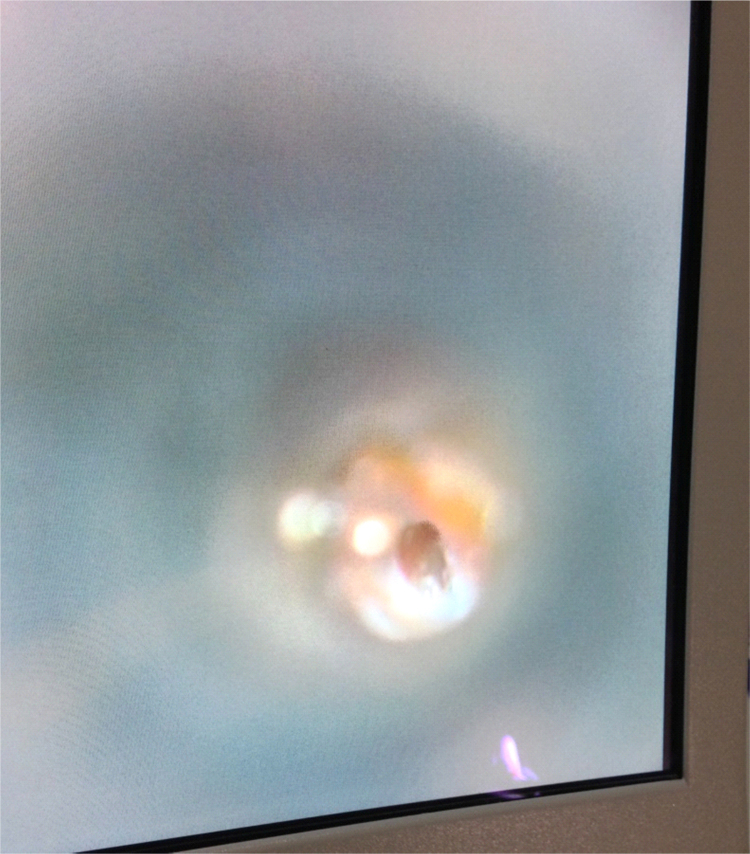
Tympanic perforation on otomicroscopy.

**Figure 2:**
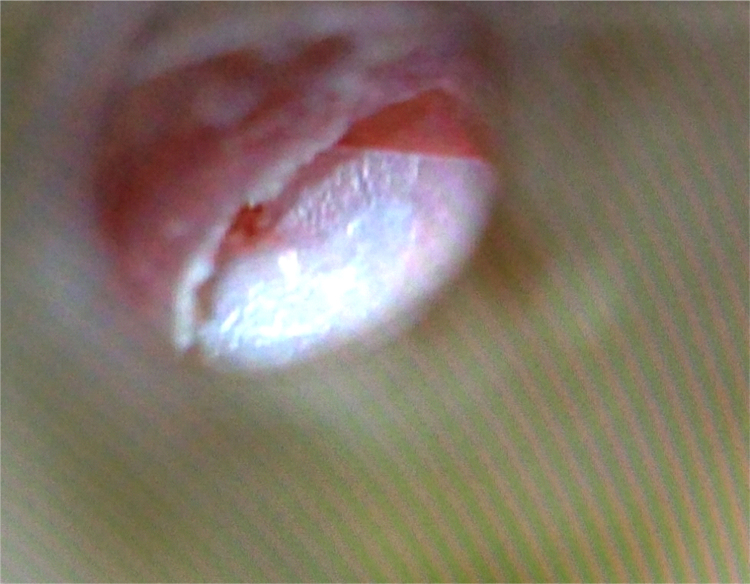
Otoendoscopy of bacterial cellulose graft over TMP.

**Figure 3:**
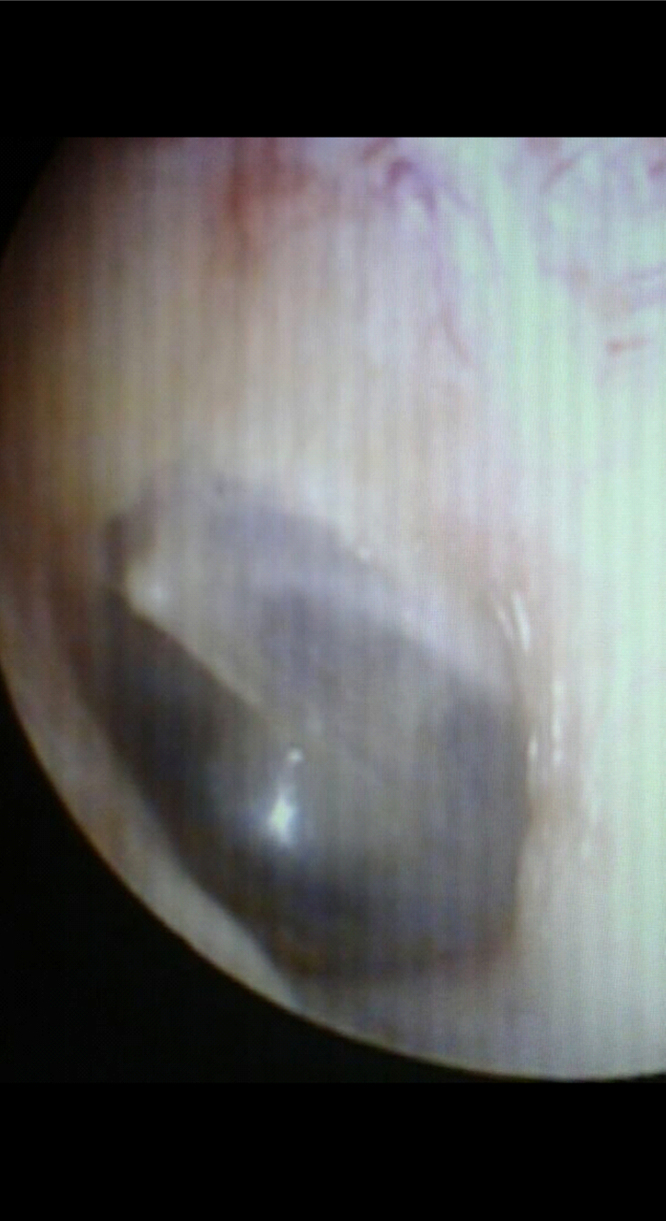
Tympanic membrane after application of bacterial cellulose graft, on microscopic examination.

Surgical time for the procedure was statistically significant (*p* < 0.001), when comparing the group that received BC graft (14.06 ± 5.23 min) *versus* autologous fascia graft (76.50 ± 17.92 min); and epithelialization time was similar in both groups, corresponding to 30 days (Table 1) (Figure 1, Figure 2, Figure 3).

**Figure 4:**
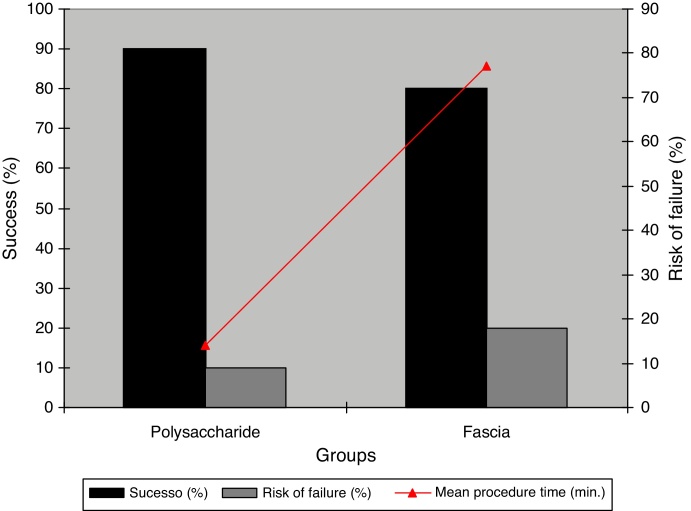
Relationship between material's success and failure *versus* procedure time.

To evaluate tympanic membrane compliance, 14 patients treated with BC graft underwent tympanometry, and of these, 13 (92.9%) had Gt within the normal range (mean Gt = 0.86 ± 0.28) and an expected At (−0.58 ± 0.28) (Fig. 4).

The relative risk (RR) of non-closure of tympanic membrane in the group treated with BC graft was lower (50%) than that for the group with autologous fascia graft. The effectiveness was 50%, a result similar for both materials (BC or fascia), despite an absolute risk reduction of 10% for TMP closure in BC group (Table 1).

## Discussion

Conventionally the treatment of TMP involves three steps: pre-operative clinical control, surgical treatment and postoperative follow-up. The main objectives of miringoplasty, in general, are to regenerate the tympanic membrane, reconstruct the sound transmission mechanism, control infection, and improve hearing. In the literature, the success rate varies from 65 to 98%.^13^, ^14^ In this study, our success rate with the use of BC membrane was 90%, compared to 80% with autologous fascia.^4^ We must also emphasize that the use of BC represented an efficiency of 50%, *i.e.* the chance of non-closure of tympanic perforation was reduced by half (RR = 0.5) *versus* temporal fascia. This is in agreement with a previous study, which followed a similar methodology and was conducted in *Chinchilla laniger*, where the authors obtained a 90% success rate.^11^

Some factors can influence the success of the surgery or of the graft, as follows: age, perforation location, size of perforation, auditory tube function, status of middle ear mucosa, type of graft used, and surgeon's experience.^15^ As to the study population, it can be added that the use of BC membrane was effective, regardless of patient's age and location and size of tympanic perforation. No adverse events related to this membrane occurred. It is noteworthy the reduction of a little more than 1 h (62.44 min) in the time required for the procedure, when comparing BC group *versus* temporal fascia (control) group, indicating that BC, in addition to being effective, shows a high level of effectiveness and practicality.

The reduction in the operating time using BC membrane is self-explanatory, because there was no need for incisions, removal of fascia, or lifting flaps. From the cost estimate for each procedure (fascia or BC membrane graft), we found a reduction of 13 times in hospital costs with the use of BC membrane; this figure represents a saving of R$ 7,178.8, considering that, with the choice of BC, there is no need for additional tests (hematology and cardiology), hospitalization or general anesthesia. The use of BC also obviates the use of special materials, such as Gelfoam™, suture material and antibiotics; on the other hand, the use of BC avoids complications such as ear pain, bleeding, and hematomas. The patient can resume immediately their daily activities.

To these aspects we can add efficiency, effectiveness and practicality, in addition to security, because this is a low-cytotoxicity and high-biocompatibility material.^6^, ^7^

As described in the literature, the tympanic membrane should be rebuilt with a connective tissue that allows for, in replacing the eardrum, ensuring its properties: elasticity, strength and ability to vibrate. Many materials have been used in the history of tympanoplasty, including free skin graft, sclera, perichondrium, temporal fascia, cartilage, and fat, among others.^16^, ^17^ It was observed that these properties were recovered, with proven data corroborated by tympanometry findings; this technique assessed tympanic membrane compliance and showed that most patients (92.9%) had Gt in the normal range and At as expected. Tympanometry, used in this study to evaluate tympanic membrane function, is a classic method applied in clinical practice, by being fast and atraumatic.^18^

Another important feature, demonstrated in previous studies, refers to BC's ability to function as an inducer of tissue remodeling and, thus, as a promoter of the healing process,^8^, ^9^, ^10^ by enabling an intensive process of revascularization^19^ and epithelialization,^11^ which may explain the regeneration of the eardrum remains and also the closure of TMP.

## Conclusion

The use of a bacterial cellulose graft promoted TMP regeneration, showing it to be an innovative, safe, efficient, effective, minimally invasive, low-cost option.

## Conflicts of interest

The authors declare no conflicts of interest.
